# The Treatment of Some Common Ailments

**Published:** 1908-01-25

**Authors:** Charles Corfield


### The General Practitioner's Column.

[Contributions to this Column are invited, and if accepted will be paid for.]

### THE TREATMENT OF SOME COMMON AILMENTS.

By CHARLES CORFIELD, M.R.C.S., L.R.C.P. (Lond.).

I remember well on first going into practice from hospital work how frequently I found that one came in contact with perplexing cases, scarcely touched on in text-books, and which never came within the purview of hospital practice at all. Since then my experience has confirmed this earlier impression, and I feel now that the general practitioner has a group of maladies quite to himself, and upon which he is the best, and perhaps the only, authority. To illustrate this I might mention influenza as an instance. Any medical man in large practice sees more of this in a period of, say, five years than does the average consultant in a lifetime. The latter no doubt has often to deal with the sequelæ, but the ordinary malady he scarcely ever sees. I should like, then, to devote a little space to the question of treating some of the common maladies, not in any sense as a claim to original work, but simply as a collection of hints and suggestions gathered from my own and others' experience. I will take first the malady of a common cold in the head. This is a very frequent trouble among one's patients, and with some febrile disturbance and malaise is sufficient to warrant advice from the family doctor. As in most other acute catarrhal affections, the patient gets at first a raw smarting feeling in the nasal passages, and with it some feeling of malaise, with headache and heaviness. Then in a day or so follows a mucous discharge, becoming later purulent. I think in these cases there is no question that nasal irrigation in some form during the earliest stages will greatly cut short the attack, and this may quite effectively be done by sniffing up from a cup or saucer. Glyco-thymolene, 1 to 3, used frequently in a nasal irrigator is a drug with which I have had considerable success in the earlier stages of this trouble. Also the well-known remedy of boracic acid and sod. bicarbonate, to which I generally add 5 or 10 grains of pot. chlor. to the ounce, is very efficacious. The bicarbonate of soda is useful in getting rid of the mucus and allowing the other drugs to act directly on the mucous membrane, disinfecting it. Then as regards the stuffiness and heaviness of the later stages of cold in the head, I generally prescribe menthol, grains twenty, dissolved in sp. camph. co., one drachm. It should be dropped on a handkerchief and inspired nasally. This prescription was suggested to me by an old family practitioner, and for the symptoms above described it certainly affords great relief.

As regards general treatment, I think this very troublesome malady is considerably aborted by putting the patient to bed for a day at least, and for the first night or so giving a dose of pulv. ipecac. co., gr. x. It is a common experience, I am sure, to find these cases when untreated linger on for weeks and lapse into a chronic condition.

Another common ailment which frequently occurs in general practice is that condition termed muscular rheumatism, although probably it has no relation to rheumatism proper at all. This will generally be noticed to come on after exposure to chill or damp following muscular "tiredness." (I emphasise the latter because, when the muscles are fresh and vigorous, exposure to similar conditions will have no effect.) It has always seemed to me that muscle is as liable as most other tissues to be subject to the effects of chill, resulting in congestion and tenderness with general febrile disturbance. As I have above suggested, the exposure is nearly always at the end of the day, and not at the beginning. This condition has been aptly called subacute muscular fibrositis. The best line of treatment is to put the patient to bed till all the muscular tenderness has disappeared—preferably, I think, between blankets—and an ordinary febrifuge prescription which aims at profuse perspiration is the most successful method of dealing with it. Possibly, though here I am on debatable ground, the muscular symptoms are due to retention of the waste products of muscle energy, and profuse diaphoresis may help to eliminate them. One drug in this malady is, I think, peculiarly useful, and that is iodide of potassium, combined with sp. am. arom. Many of these cases manifest in particular, severe lumbago, which is often very intractable. I have found dry-cupping very useful in such instances, and as it is exceedingly simple to do it should always be tried. It may be repeated daily for three or four days. Another method from which I have often had successful results is to paint the lumbar area with iodine, and then apply over the same surface a hot iron with flannel interposed.

Another trouble of considerable interest to general practitioners is varicose ulcer of the leg, which is often very obstinate to treatment. Most frequently it is dressed with ointment spread on lint, and then bandaged. Now lint well smeared with ointment scarcely allows any outlet for the surface discharge. By spreading the ointment on gauze, over which is placed a pad of wool, all the discharge is absorbed, the surface of the ulcer is kept clean, and the specific action of the ointment is allowed full play. To this treatment, combined with firm bandaging, I have seen many obstinate cases succumb, and ultimately clear up.

There is another group of conditions which, included under the name of sepsis, are unfortunately often met with in general practice. In septic, as in all other maladies, there are the two factors at work—namely, predisposing and exciting, or, perhaps, in simpler language, the constitutional and the local. For instance, a lowered vitality, weak cell resistance, coupled with a pin prick frequently means a whitlow; or a scratch or abrasion a diffuse and virulent cellulitis. We all know that active treatment must be adopted in such conditions, and, to sum it up briefly, this should be free incisions and large doses of liq. ferri perchlor. Now as regards local treatment, I may say that the general practitioner, either from timidity or from the fact that he is unable to let the patient have a general anæsthetic, scarcely ever incises boldy enough. Many times while in charge of the septic ward in my hospital days I had cases sent in of cellulitis in its various forms, with often perhaps one small, almost punctured incision. The result being that there is no outlet for the sero-purulent exude, which, causing extreme tension, brought about sloughing as well as toxic absorption from the pent-up pus.

In all these cases the patient should have a general anæsthetic, gas or a few whiffs of chloroform being usually quite sufficient, and the inflamed area freely incised and drained. Much hæmorrhage usually follows, which, however, is rarely troublesome. The part should then have frequent and prolonged immersion in a warm boracic bath.

As regard the general treatment, large doses of liq. ferri are almost specific. The great objection that this drug is not readily tolerated in the stomach is easily overcome by free dilution. Thus: liq. ferri ♏ xxx, glycerin. ♏ xxx, aq. chlorof. ad one ounce. To be given every two or three hours in half a tumbler of water. I am quite aware that this form of iron is therapeutically supposed not to be absorbed, but clinical experience of a great number of septic cases only convinces me more fully that it has a very marked action on the course of the disease, bringing down the temperature, and effecting a decided improvement in the general condition.

